# Deciphering network dysregulations and temporo-spatial dynamics in disorders of consciousness: insights from minimum spanning tree analysis

**DOI:** 10.3389/fpsyg.2024.1458339

**Published:** 2024-12-19

**Authors:** Yangyang Dai, Qiheng He, Shan Wang, Tianqing Cao, Xiaoke Chai, Nan Wang, Yijun Dong, Peiling Wong, Jianghong He, Feng Duan, Yi Yang

**Affiliations:** ^1^Tianjin Key Laboratory of Brain Science and Intelligent Rehabilitation, College of Artificial Intelligence, Nankai University, Tianjin, China; ^2^Department of Neurosurgery, Beijing Tiantan Hospital, Capital Medical University, Beijing, China; ^3^Department of Information and Communications Engineering, School of Engineering, Tokyo Institute of Technology, Yokohama, Kanagawa, Japan; ^4^Chinese Academy of Medical Sciences and Peking Union Medical College, Beijing, China; ^5^Department of Physical Therapy and Assistive Technology, National Yang Ming Chiao Tung University, Taiwan, China; ^6^Chinese Institute for Brain Research, Beijing, China; ^7^Beijing Institute of Brain Disorders, Beijing, China; ^8^China National Clinical Research Center for Neurological Diseases, Beijing, China

**Keywords:** disorders of consciousness, minimum spanning tree, static functional connectivity, dynamic functional connectivity, consciousness-related neural mechanism

## Abstract

**Objectives:**

The neural mechanism associated with impaired consciousness is not fully clear. We aim to explore the association between static and dynamic minimum spanning tree (MST) characteristics and neural mechanism underlying impaired consciousness.

**Methods:**

MSTs were constructed based on full-length functional magnetic resonance imaging (fMRI) signals and fMRI signal segments within each time window. Global and local measures of static MSTs, as well as spatio-temporal interaction characteristics of dynamic MSTs were investigated.

**Results:**

A disruption or an alteration in the functional connectivity, the decreased average coupling strength and the reorganization of hub nodes were observed in patients with minimally conscious state (MCS) and patients with vegetative state (*VS*). The analysis of global and local measures quantitatively supported altered static functional connectivity patterns and revealed a slower information transmission efficiency in both patient groups. From a dynamic perspective, the spatial distribution of hub nodes exhibited relative stability over time in both normal and patient populations. The increased temporal variability in multiple brain regions within resting-state networks associated with consciousness was detected in MCS patients and *VS* patients, especially thalamus. As well, the increased spatial variability in multiple brain regions within these resting-state networks was detected in MCS patients and *VS* patients. In addition, local measure and spatio-temporal variability analysis indicated that the differences in network structure between two groups of patients were mainly in frontoparietal network and auditory network.

**Conclusion:**

Our findings suggest that altered static and dynamic MST characteristics may shed some light on neural mechanism underlying impaired consciousness.

## 1 Introduction

Brain injuries affect nearly half of the global population at some point in their lives (Jiang et al., 2019). Disorder of consciousness (DoC) (Fernandez-Espejo and Owen, 2013; Bernat, 2006) is a neurological dysfunction caused by severe brain injury which results in the inability of the body to recognize its surroundings and its own state in a continuous and organized manner. This condition may progress from a coma to a vegetative state (*VS*), a minimally conscious state (MCS), and eventually an emergence from MCS. The post-treatment and care of DoC patients remain significant challenges in the medical field.

Despite extensive research, the neural mechanism underpinning consciousness is not fully understood. Advancements in neuroimaging techniques and complex network theory have highlighted new possibilities for extracting consciousness-related biomarkers in DoC patients. Cognitive functions emerge from interactions between various brain regions. Complex network theory abstracts these regions and their interactions into nodes and connection edges, respectively. Abundant evidence suggests that DoC is associated with abnormal changes in brain networks (Crone et al., 2014; Rizkallah et al., 2019; Tan et al., 2019). The brain is dynamically changing. Dynamic brain connectivity is able to capture the time-varying characteristics of information interactions in the brain, compensating for the limitations of static brain connectivity. Recently, researchers have widely investigated on the dynamic brain connectivity associated with DoC patients using different approaches. The dynamic and time-resolved functional connectivity studies reports that there is a significant difference in the mean dwell time and number of transitions between the normal population and DoC patients (Cao et al., 2019), and *VS* patients exhibits decreased dwell time and loss of non-stationarity in default mode network (DMN) and subcortical frontoparietal-temporal network compared to MCS patients(Panda et al., 2022). This suggests that the network flexibility is reconfigured under the impact of DoC (Cai et al., 2020). A study based on a spatio-temporal causal interaction model points to a decreased ability in neural propagation and response to events in DoC patients (Panda et al., 2023). The authors believe disruption of information transmission in posterior cortical regions, along with reduced information broadcasting in subcortical, temporal, parietal, and frontal regions may contribute to loss of consciousness. Another study uses a whole-brain model based on a supercritical Hopf bifurcation (Lopez-Gonzalez et al., 2021; Perl et al., 2023) to interpret the dynamical effects that DoC patient exhibits less recurrent, less connected and more segregated synchronization patterns. Also, the turbulence approach (Escrichs et al., 2022) is introduced to examine dynamic brain connectivity in DoC patients.

Traditional network construction heavily relies on threshold selection, lacking a clear method to filter out spurious connections. The minimum spanning tree (MST) is an acyclic subgraph extracted from the original network, connecting all nodes with the minimum overall weight (Tewarie et al., 2015). MST is unique and represents the crucial skeleton structure of the original network. MST-based analysis avoids the threshold selection problem, standardizing the construction of brain networks. MST is vital for global network transmission (Van Mieghem and Magdalena, 2005; Van Mieghem and van Langen, 2005; Wang et al., 2008). Studies have substantiated that neuropsychiatric disorders lead to the disruption of the MST structure (Liu et al., 2021; Wang et al., 2018; Stam et al., 2014). However, there is a gap in understanding how impaired consciousness affects the MST structure. More, exploring the spatio-temporal interaction of dynamic MSTs in relation to consciousness becomes imperative.

This study constructs static and dynamic MSTs using resting-state functional magnetic resonance imaging (fMRI) signals acquired from 30 healthy subjects, 21 MCS patients and 66 *VS* patients. Then, static and dynamic MST characteristics are calculated for each group. Our main aim is to capture the abnormal brain activities induced by DoC through the altered network characteristics in an attempt to explain the neural mechanism behind impaired consciousness.

## 2 Materials and methods

### 2.1 Subjects

This study included a total of 140 DoC patients from the Department of Neurosurgery, Beijing Tiantan Hospital, Capital Medical University. Each patient had received a severe brain damage for more than 1 month. All patients were assessed multiple times during the 2 weeks prior to baseline based on the Coma Recovery Scale-Revised. It was ultimately determined that 113 of these patients were in a *VS* and 27 were in an MCS. The fMRI data from 6 MCS patients and 47 *VS* patients were discarded due to brain damage exceeding 30% of the entire brain volume, excessive head movements, or poor image quality. Clinical details for the remaining 21 MCS patients (age = 40.14 ± 14.16, 13 male) and 66 *VS* patients (age = 41.42 ± 14.77, 40 male) are provided in Supplementary Table S1. 30 healthy subjects (age = 41.24 ± 11.27, 15 male) were recruited into the normal control (NC) group, ensuring age and gender matching. None had a history of neurological or psychiatric diseases. The Ethics Committee of Beijing Tiantan Hospital approved this study, and informed consent was obtained from each healthy volunteer and legal surrogate of each DoC patient (KY2023-161). During data acquisition, no sedative or anesthetic drugs were injected into the patients. Partial fMRI data have been used in our previous studies (Yang et al., 2023).

### 2.2 Data acquisition and preprocessing

A Discovery MR750 3.0-T scanner (General Electric, Milwaukee, Wisconsin, USA) was used to collect all images. fMRI images were obtained with the following parameters, volumes = 210, repetition time (TR)/echo time = 2000/30 ms, 39 slices, thickness/gap = 4.0/0.6 mm, voxel size = 3.75 × 3.75 × 4 mm^3^, flip angle = 90^°^, matrix size = 64 × 64, field of view = 240 × 240 mm. T1-weighted high-resolution structural images were also scanned so as to examine whether there were large brain aberrations or focal brain damage in the patients.

Data preprocessing, performed using Data Processing & Analysis for Brain Imaging Version 6.0210501 software package (Yan et al., 2016), included discarding the first 10 images, slice timing correction, realignment, co-registration of the functional images to the standard space of Montreal Neurological Institute, smoothing with a 4-mm full-width at half maximum Gaussian kernel, regression of nuisance signals and head motion parameters, and temporal filtering (0.01–0.1 Hz). Excessive head motion severely affects the quality of the signal, thus introducing complicated variations in the subsequent static and dynamic MST analysis. Therefore, framewise displacement >0.5 mm exceeding 50% led to the discarding of corresponding fMRI data.

### 2.3 The construction and analysis of static MSTs

The Automated Anatomical Labeling (AAL) atlas, parcellating the brain into 90 regions, was used to construct brain functional networks. Brain regions were mapped as nodes, each associated with a time series obtained by averaging time series of all voxels within the corresponding brain region. The coupling relationship between node pairs was measured by Pearson’s correlation coefficients, corrected by a Fisher’s *r*-to-*z* transformation. The static MSTs were computed from the static functional connectivity matrices, involving tasks such as discarding the negative elements and converting the connection weights to their reciprocals. Three local (degree, betweenness centrality, eccentricity) and four global (characteristic path length, diameter, leaf fraction, tree hierarchy) indicators were included to catch static MST characteristics in normal subjects and DoC patients.

The degree of a node is defined as the total number of all edges connected to that node. The Equation 1 is expressed as follows


(1) $$k_{i}=\sum_{j\in V,j\neq i}a_{ij}$$

where $k_{i}$ denotes the degree of node i, $a_{ij}$ denotes the link between node i and node j, V denotes the set of nodes.

Betweenness centrality is the proportion of shortest paths between all node pairs through a given node, which emphasizes the importance of a node in communication control. The Equation 2 is expressed as follows


(2) $$BC_{i}=\frac{1}{\left(N-1\right)\left(N-2\right)}\sum_{h,i,j\in V;h\neq i,h\neq j,i\neq j}\frac{\rho_{hj}^{i}}{\rho_{hj}}$$

where $BC_{i}$ represents the betweenness centrality of node i, $\rho_{hj}^{i}$ represents the number of the shortest path between node h and node j passing through node i, $\rho_{hj}$ represents the number of the shortest path between node h and node j, N represents to the total number of nodes.

The eccentricity of a node refers to the maximum value of the shortest path length from a given node to other nodes. The Equation 3 is as follows


(3) $$Ecc_{i}=\max\left\{d\left(i,j\right)|j\in V\right\}$$

where $Ecc_{i}$ refers to the eccentricity of node i, $d\left(i,j\right)$ refers to the shortest path from node i to node j.

The characteristic path length is obtained by calculating the average shortest path length of all node pairs. The Equation 4 is expressed as follows.


(4) $$Lp=\frac{1}{N\left(N-1\right)}\sum_{i\neq j}d\left(i,j\right)$$

where Lp stands for characteristic path length.

The diameter of an MST is the longest shortest path length of all node pairs, or the maximum eccentricity. The Equation 5 is listed below


(5) $$D=\max\left\{Ecc_{i}|i\in V\right\}$$

where D indicates the diameter.

The leaf fraction indicates the rate of leaf nodes, which have only one link. The Equation 6 is shown as follows


(6) $$Lf=\frac{L}{M}$$

where Lf denotes the leaf fraction, L denotes the number of leaf nodes, M denotes the number of links.

The tree hierarchy was introduced to quantify information integration in and overload protection. The Equation 7 is listed as follows


(7) $$T_{H}=\frac{L}{2MBC_{\text{max}}}$$

where $T_{H}$ represents the tree hierarchy, $BC_{\text{max}}$ represents the maximum betweenness centrality. The tree hierarchy varies from 0 to 1. In a line-like MST, L=2. With M progressively approaching infinity, the tree hierarchy becomes closer to 0. In a star-like MST, L≈M, so the tree hierarchy is closer to 0.5. In a hierarchical MST, the tree hierarchy trends to 1. For more details on a star-like MST, a line-like MST, and a hierarchical MST, refer to the literatures (He et al., 2019; Blomsma et al., 2022; Tewarie et al., 2014).

### 2.4 The construction and analysis of dynamic MSTs

The dynamic brain functional network was estimated using a sliding window approach. A fixed-length time window (50 TR) intercepted signals along the time axis in a sliding step of 1 TR. Pearson’s correlation coefficients characterized the coupling relationships between the signal segments within each window, thus generating 151 functional connectivity matrices per subject. Likewise, these matrices underwent nonlinear correction with a Fisher’s *r*-to-*z* transformation.

Dynamic MSTs were derived from coupling relationships between signal segments within each time window, akin to static MST construction. Nodal degree was employed to identify hubs within each time window, exploring dynamic variability. Considering the brain’s inherent spatio-temporal interactions, temporal and spatial variability (Chu et al., 2023; Zhang et al., 2016) were introduced to examine altered neural activities in the context of impaired consciousness.

Assume that $F=\left\{F_{1},\cdots ,F_{r},\cdots ,F_{v}\right\}$ is the real functional connectivity architecture within v time windows derived from the dynamic MSTs. The temporal variability of a specific brain region can be calculated according to Equation 8


(8) $$T_{i}=1-\frac{1}{v\left(v-1\right)}\sum_{p,q=1;p\neq q}^{v}\text{corr}\left(F_{p}\left(i,:\right),F_{q}\left(i,:\right)\right)$$

where $F_{p}\left(i,:\right)$ reflects the coupling relationship between brain region i and other brain regions within the *p*th time window. Apparently, so is $F_{q}\left(i,:\right)$. $\text{corr}\left(F_{p}\left(i,:\right),F_{q}\left(i,:\right)\right)$ quantifies the temporal similarity in the functional connectivity architecture of brain region i within two different time windows.

The spatial functional connectivity sequence $F_{s}\left(i,j\right)={\left[F_{1}\left(i,j\right),F_{2}\left(i,j\right),\cdots ,F_{v}\left(i,j\right)\right]}^{T}$ describes the changing profile of the coupling relationship between brain region i and brain region j over all time windows. The spatial variability of brain region *i* is expressed as Equation 9


(9) $$S_{i}=1-\frac{1}{\left(N-1\right)\left(N-2\right)}\sum_{j,h=1;j\neq h\neq i}^{N}\text{corr}\left(F_{s}\left(i,j\right),F_{h}\left(i,h\right)\right)$$

where N denotes the number of brain regions. $\text{corr}\left(F_{s}\left(i,j\right),F_{h}\left(i,h\right)\right)$ reveals the spatial similarity of two different spatial functional connectivity sequences associated with brain region i.

**Figure 1:**
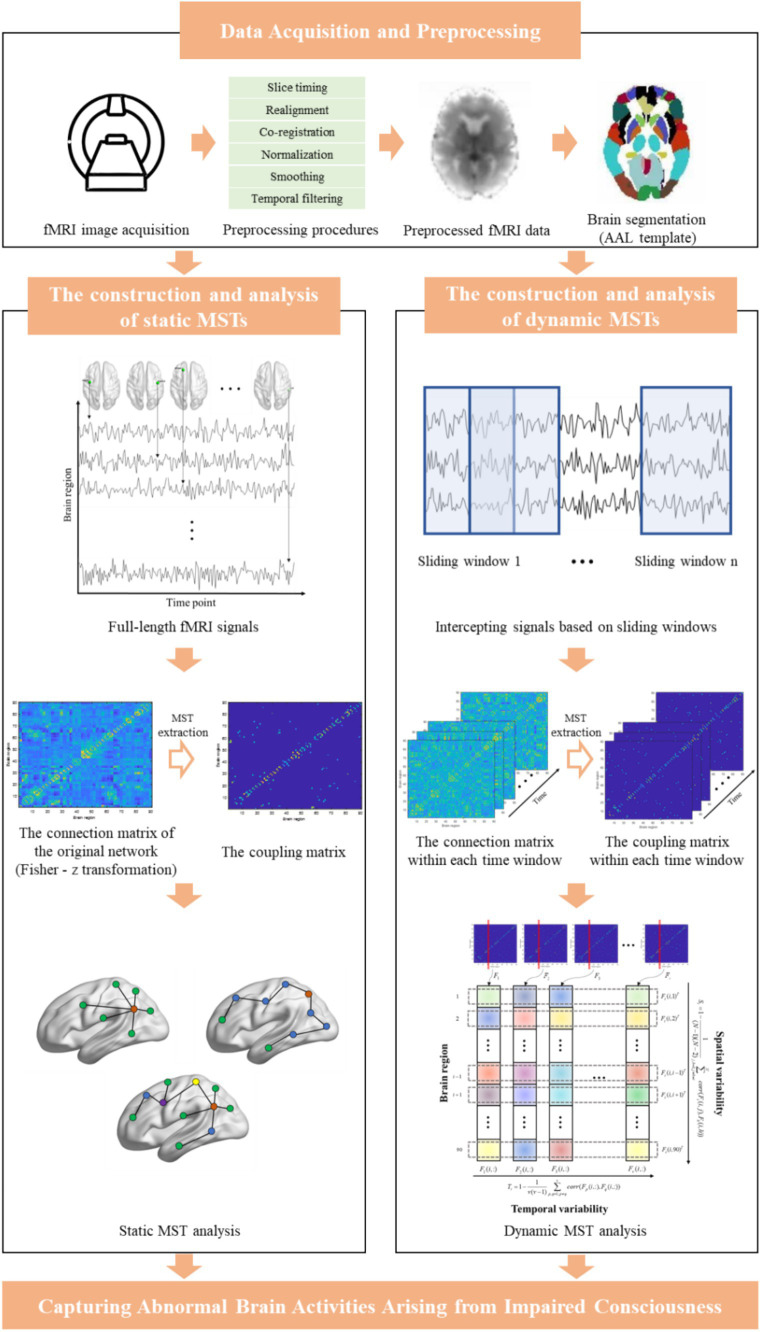
The main framework of this study.

The main framework of the current study is summarized in Figure 1.

### 2.5 Statistical analysis

In this study, a nonparametric permutation test with 10,000 permutations (significance level 0.05) was employed to detecting statistical differences in static and dynamic characteristics between the normal population, MCS patients and *VS* patients. In addition, a false discovery rate (FDR) algorithm (significance level 0.05) was introduced to control the effects of multiple comparisons.

## 3 Results

### 3.1 Average coupling strength

**Figure 2:**
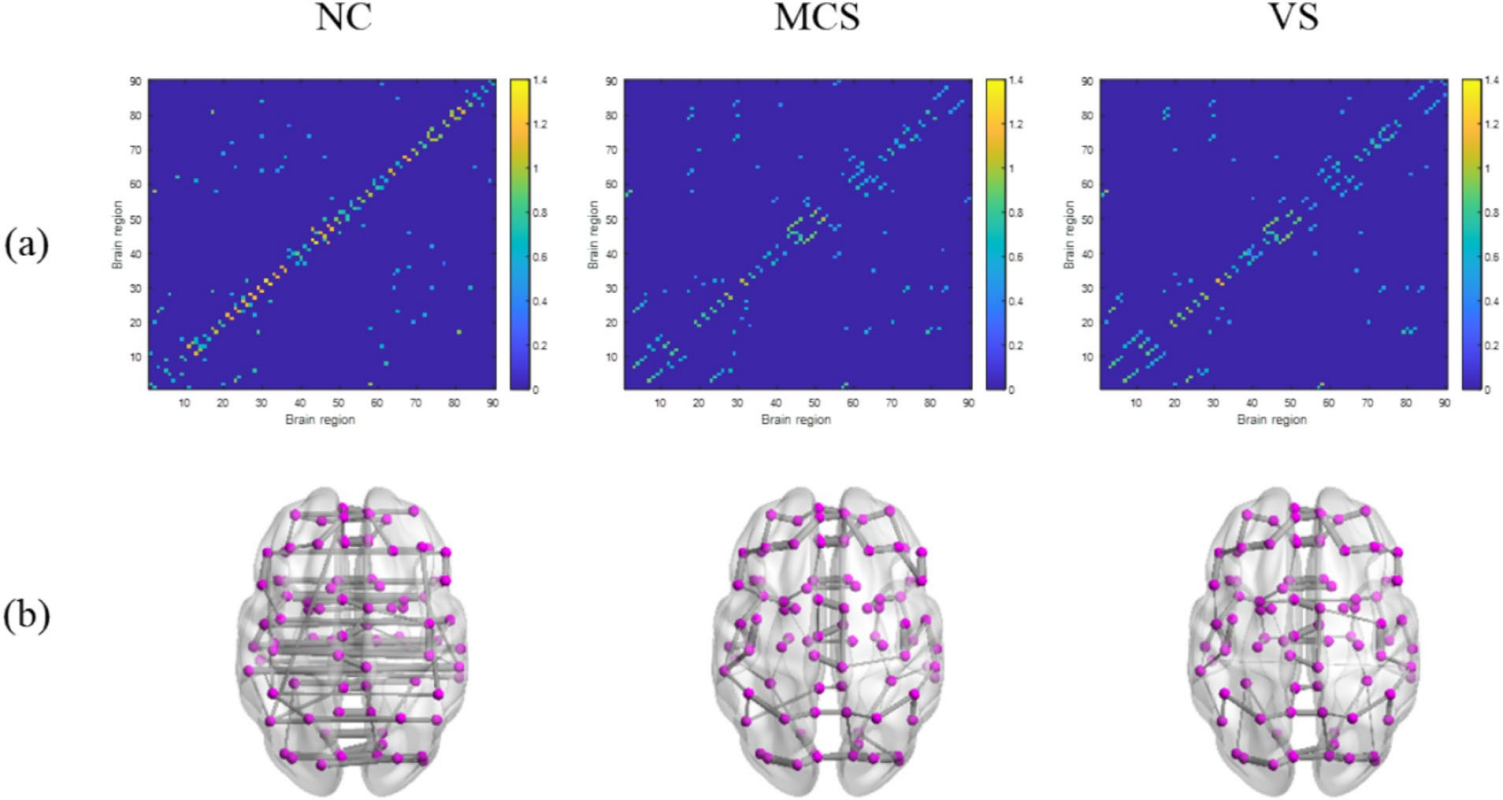
The node coupling relationship between brain regions and the critical network structures extracted based on static MSTs. **(A)** The connection matrix; **(B)** the brain functional network. In **(A,B)**, the connection matrices and the network structures extracted based on static MSTs are shown from left to right for the normal population, MCS patients, and *VS* patients, respectively.

The coupling strength of node pairs quantifies the interdependencies between brain regions, as expressed by the transformed Pearson’s correlation coefficient of the corresponding time series. The node coupling relationship of static MSTs extracted from the average functional connection matrices of the normal population, MCS patients and *VS* patients was shown in Figure 2A. We found a decrease in the average coupling strength of MCS patients and *VS* patients compared with normal individuals (*p* < 0.05, FDR corrected), whereas there was no difference in the average coupling strength between both groups (*p* > 0.05). This suggests that DoC may induce a weaker interdependence between brain regions. Further, the connection matrix was mapped into glass brain for better visualization, as shown in Figure 2B. Here, a disruption or an alteration in the functional connectivity of MCS patients and *VS* patients was intuitively observed.

### 3.2 Static MST topology and critical brain regions

**Figure 3:**
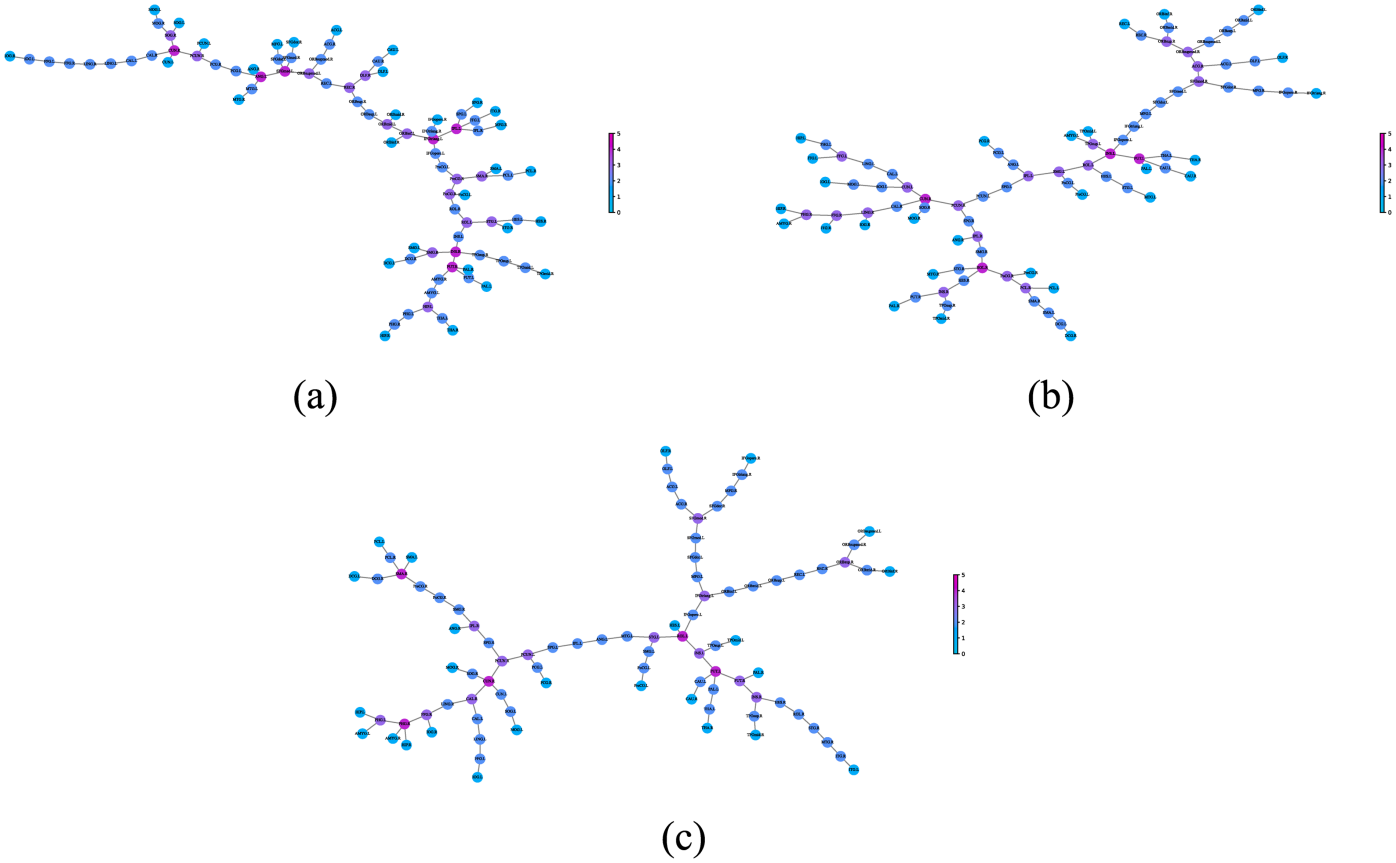
The topological structure of static MST in each group. **(A)** NC group; **(B)** MCS group; **(C)** *VS* group. The static MST topology of the normal population and patients is an intermediate configuration between a line-like and a star-like MST structure. A node, whose degree is at least one standard deviation greater than the mean, is considered as a hub node. In figure, the nodes with color close to fuchsia have more links and can be considered as hub nodes.

Using the connection pattern illustrated in Figure 2, we plotted the static MST topology for each group in Figure 3. It can be seen that, as in the normal population, the static MST topology of MCS patients and *VS* patients remains an intermediate configuration between a line-like and a star-like MST structure. This will be further elaborated in the analysis of the global measures.

A highly connected node is usually considered to be a hub node of the network. In Figure 3, we depict the nodes with different colors and sizes in accordance with their degree. Nodes, labeled by AAL atlas abbreviations (provided in Supplementary Table S2), were scrutinized to identify the critical brain regions (generally its degree at least one standard deviation greater than the mean). Preliminarily, there were differences in the hub nodes not only between the normal population and the patients, but also between the MCS patients and the *VS* patients.

### 3.3 Global measures

**Figure 4:**
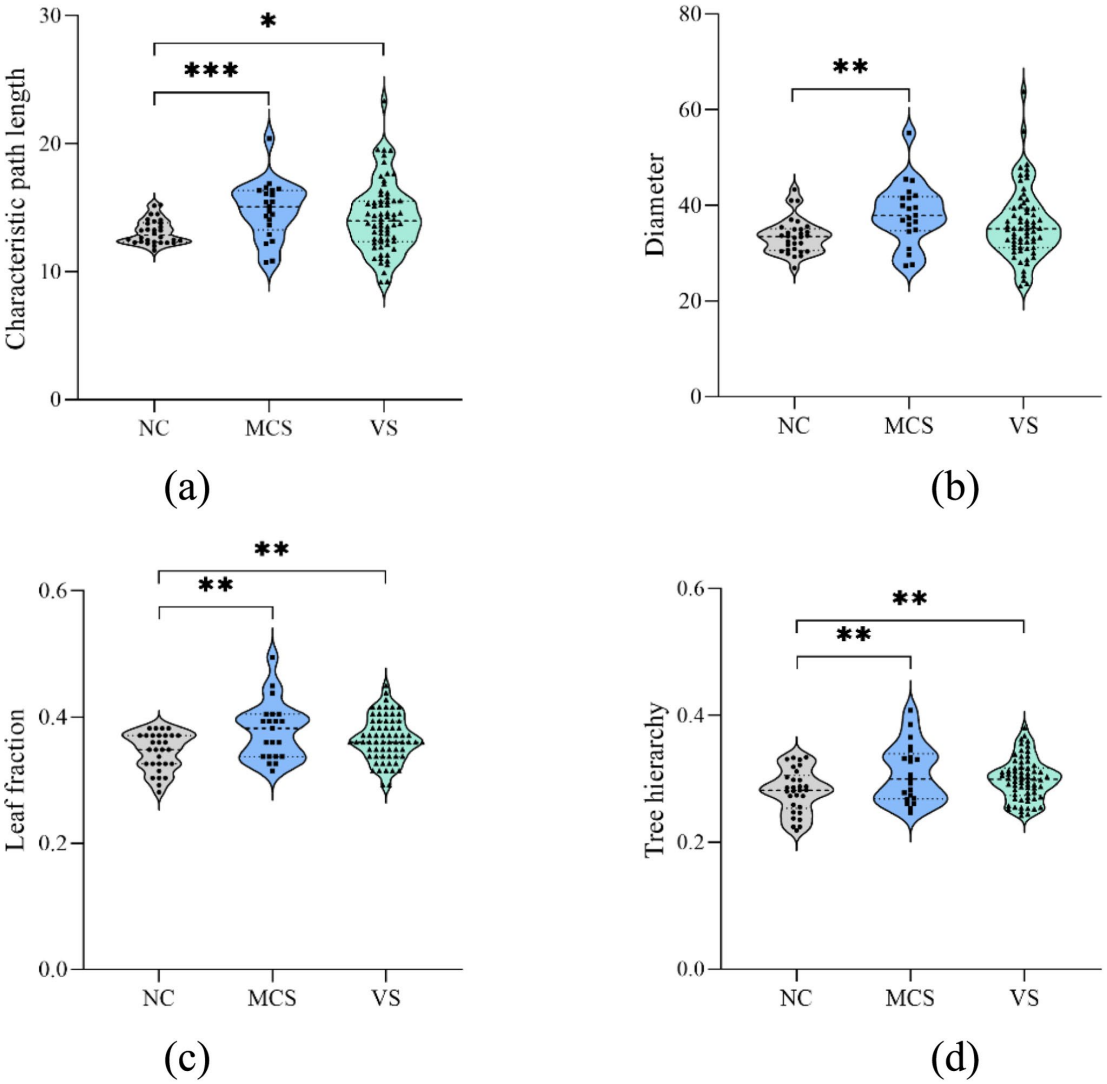
Between-group differences in static MST global measures. **(A)** Characteristic path length; **(B)** diameter; **(C)** leaf fraction; **(D)** tree hierarchy. The increased characteristic path length, leaf fraction and tree hierarchy were observed in MCS patients and *VS* patients. The increased diameter was detected in MCS patients. *p < 0.05, FDR corrected; **p < 0.01, FDR corrected; *** *p* < 0.001, FDR corrected.

To delve deeper into the impact of DoC, global measure changes in static MSTs were validated (Figure 4). The significant between-group differences were emphasized with asterisks. Compared to the normal population, 4 global measures were increased in MCS patients (*p* < 0.05, FDR corrected). The characteristic path length, leaf fraction and tree hierarchy were increased in *VS* patients (*p* < 0.05, FDR corrected), but diameter of *VS* patients was not different from that of the normal population (*p* > 0.05, FDR corrected). No difference was found between MCS patients and *VS* patients for each global measure (*p* > 0.05).

### 3.4 Local measures

**Figure 5:**
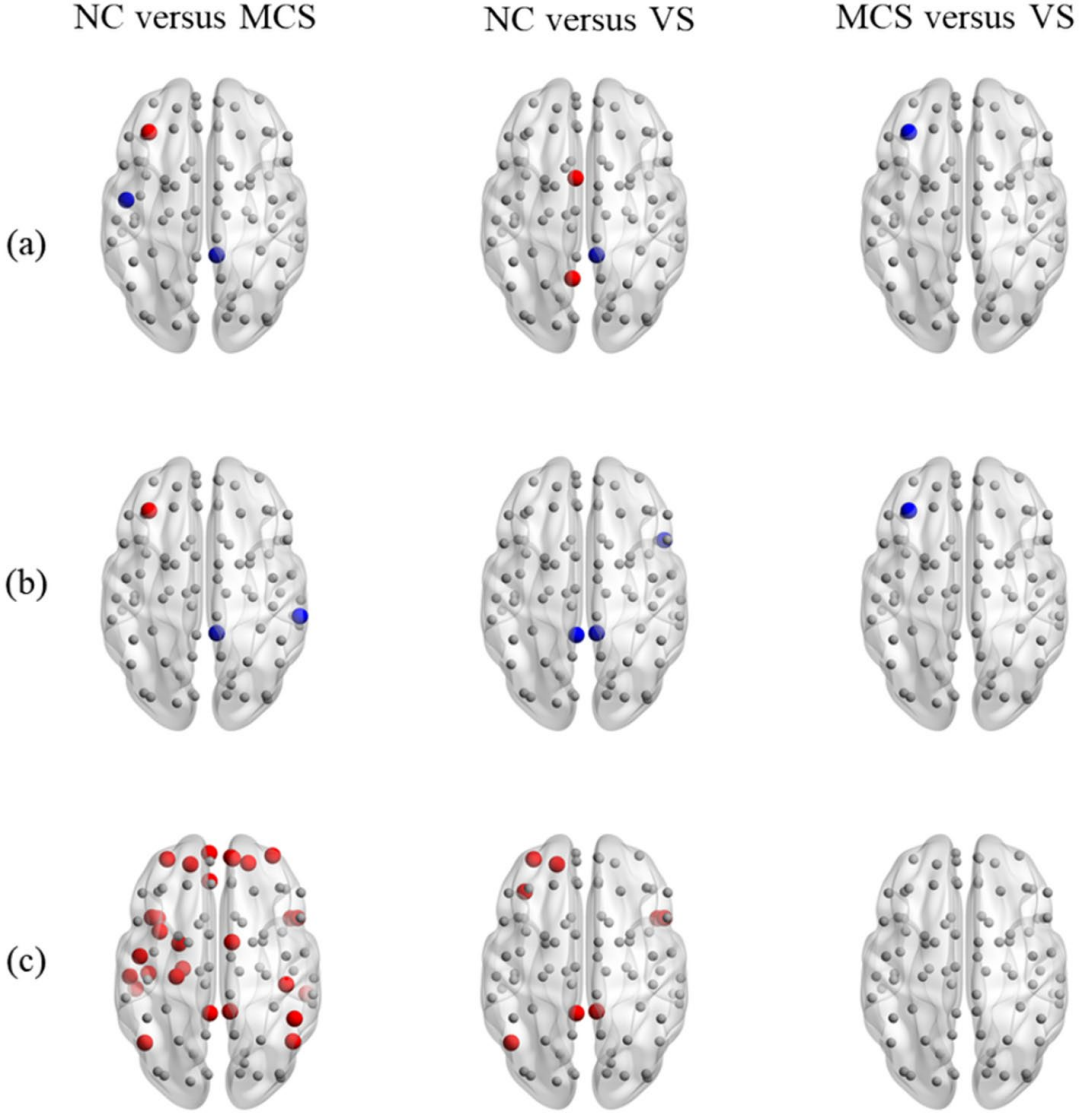
Between-group differences in static MST lobal measures. **(A)** Degree; **(B)** betweenness centrality; **(C)** eccentricity. In figure, from left to right, between-group comparison of local measures is shown for the normal population and MCS patients, the normal population and *VS* patients, as well as MCS patients and *VS* patients. The brain regions with decreased local measures (MCS < NC, *VS* < NC, and *VS* < MCS) are marked with larger blue nodes. The brain regions with increased local measures (MCS > NC, *VS* > NC, and *VS* > MCS) are marked with larger red nodes. The brain regions with no significant differences in local measures are marked with smaller gray nodes.

The examination of local measures highlighted the subtle changes in brain activities of some specific brain regions caused by DoC, as illustrated in Figure 5. In Figure 5, the brain regions with no significant differences in local measures were marked with the smaller gray nodes. The brain regions with increased and decreased local measures were emphasized by the larger red and blue nodes, respectively. Compared to the normal population, the brain regions located in frontoparietal network (FPN), DMN, and auditory network (AUN) were detected to have an increased or decreased degree in MCS patients. The brain regions located in DMN and sensorimotor network (SMN) were detected to have an increased or decreased degree in *VS* patients. The brain regions located in FPN, AUN and DMN were detected to have an increased or decreased betweenness centrality in MCS patients. The brain regions located in cingulo-opercular network (CON) and DMN were detected to have a decreased betweenness centrality in *VS* patients. The brain regions mainly located in FPN, AUN, SMN, DMN, CON, and salience network (SN) were detected to have an increased eccentricity in MCS patients. The brain regions located in CON, FPN, DMN and subcortical network (SCN) were detected to have an increased eccentricity in *VS* patients. As for both groups of patients, the brain regions located in FPN were detected with decreased degree and betweenness centrality in *VS* patients. No difference in eccentricity was observed between the brain regions of MCS patients and those of *VS* patients. More details on the changes in local measures were summarized in Supplementary Tables S3.

### 3.5 Dynamic variability of MST hubs

**Figure 6:**
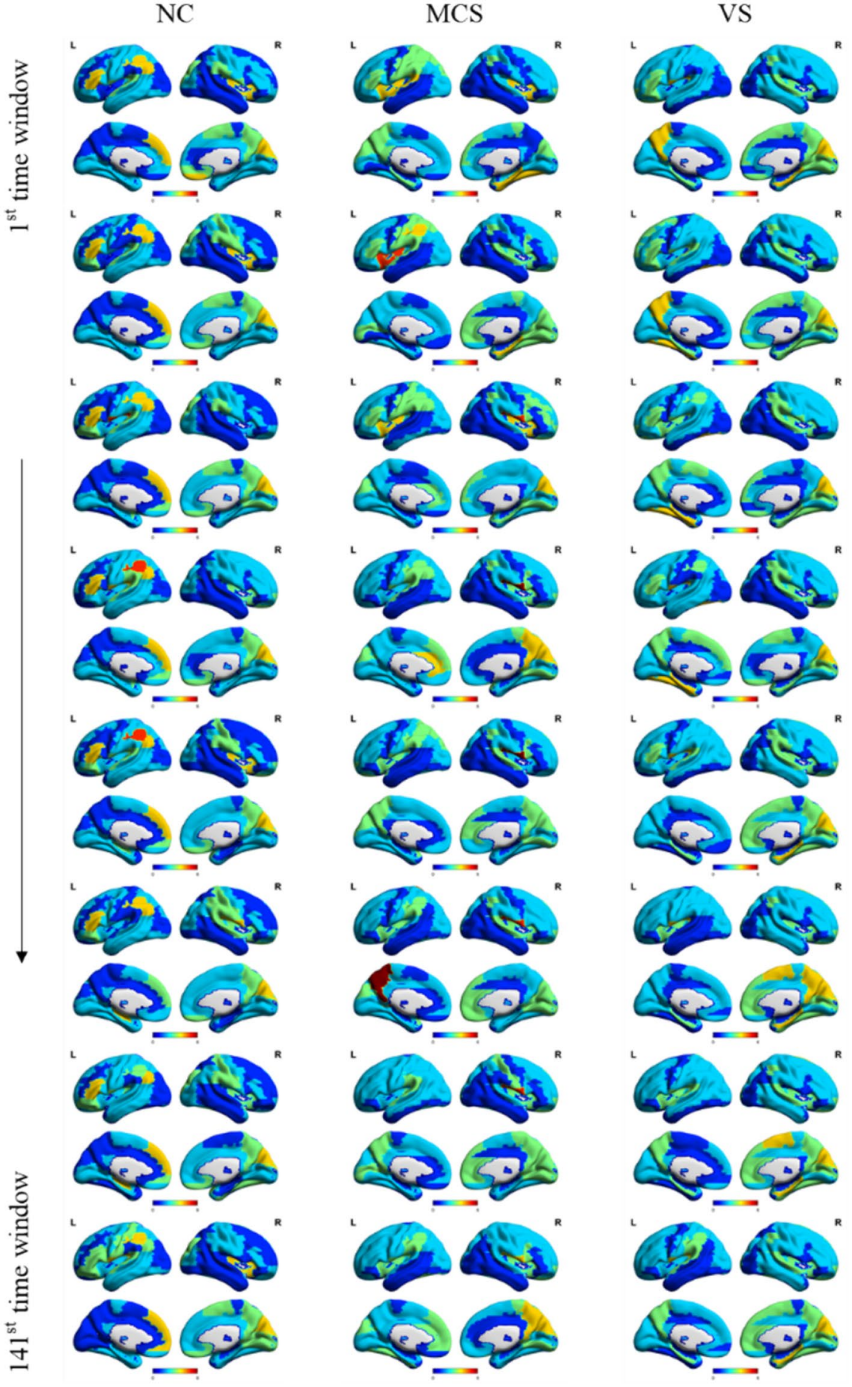
The stability of MST Hub nodes. The spatial distribution of hub nodes was plotted within 8 different time windows in an interval of 20 for the normal population, MCS patients and *VS* patients.

The hub nodes of the human brain play a crucial role in the information transmission between different brain regions. However, we still know little about the time-varying properties of hub nodes. Here, nodal degree within different time windows, mapped to the brain surface using the BrainNet Viewer (Xia et al., 2013) software package, provided insights into the spatial distribution of hub nodes over time. Figure 6 showed the spatial distribution of hub nodes in the normal population, MCS patients and *VS* patients within 8 different time windows in an interval of 20. The spatial distribution of hub nodes within each window was provided in Supplementary Figure S1. Surprisingly, the spatial distribution of hub nodes was relatively stable over time in each group.

### 3.6 Temporal and spatial variability analysis

**Figure 7:**
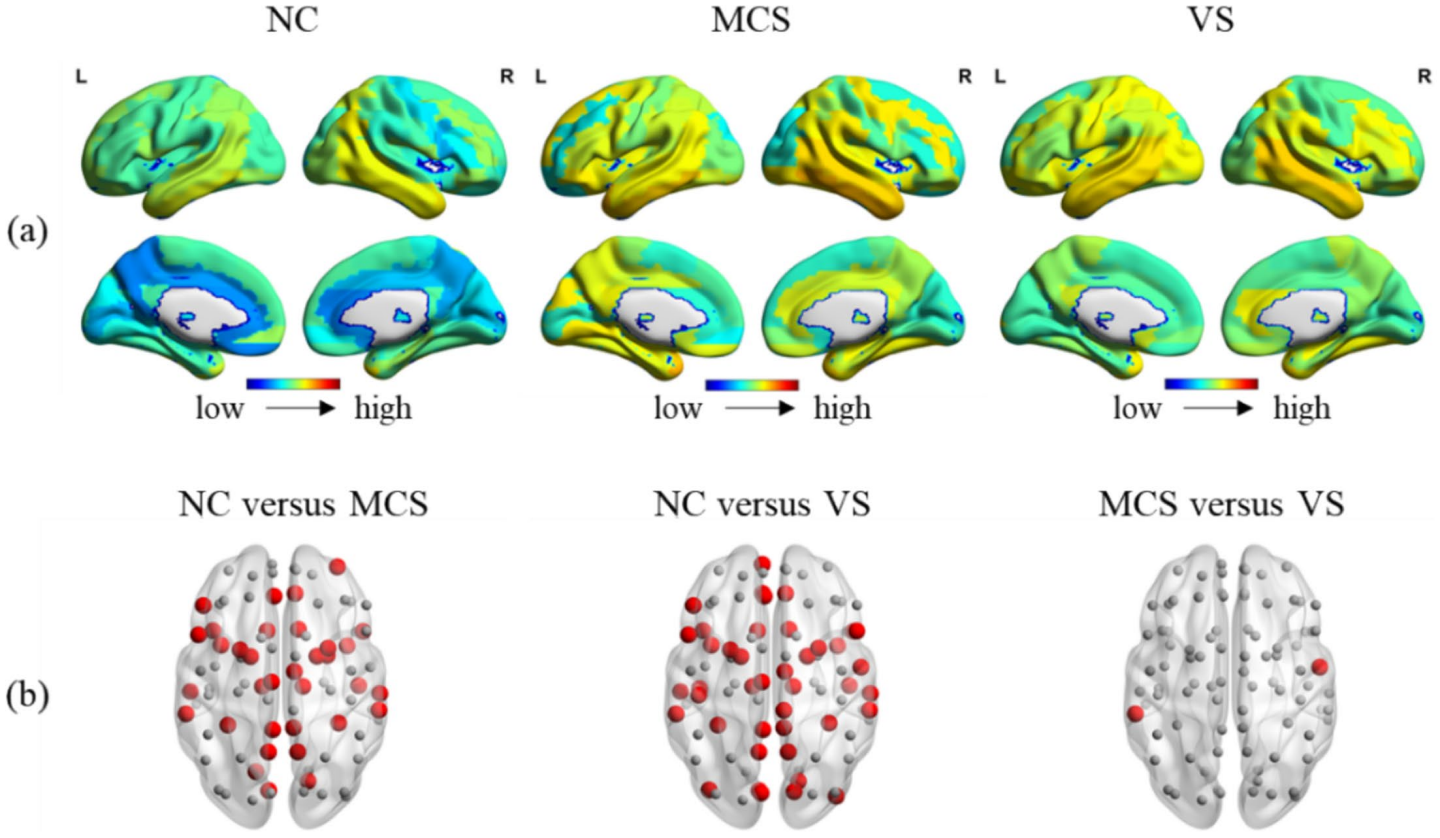
Between-group comparison in temporal variability. **(A)** The temporal variability across brain regions in the normal population, MCS patients and MCS patients; **(B)** between-group comparison in temporal variability. The brain regions with increased temporal variability (MCS > NC, *VS* > NC, and *VS* > MCS) are marked with larger red nodes. The brain regions with no significant differences in temporal variability are marked with smaller gray nodes.

**Figure 8:**
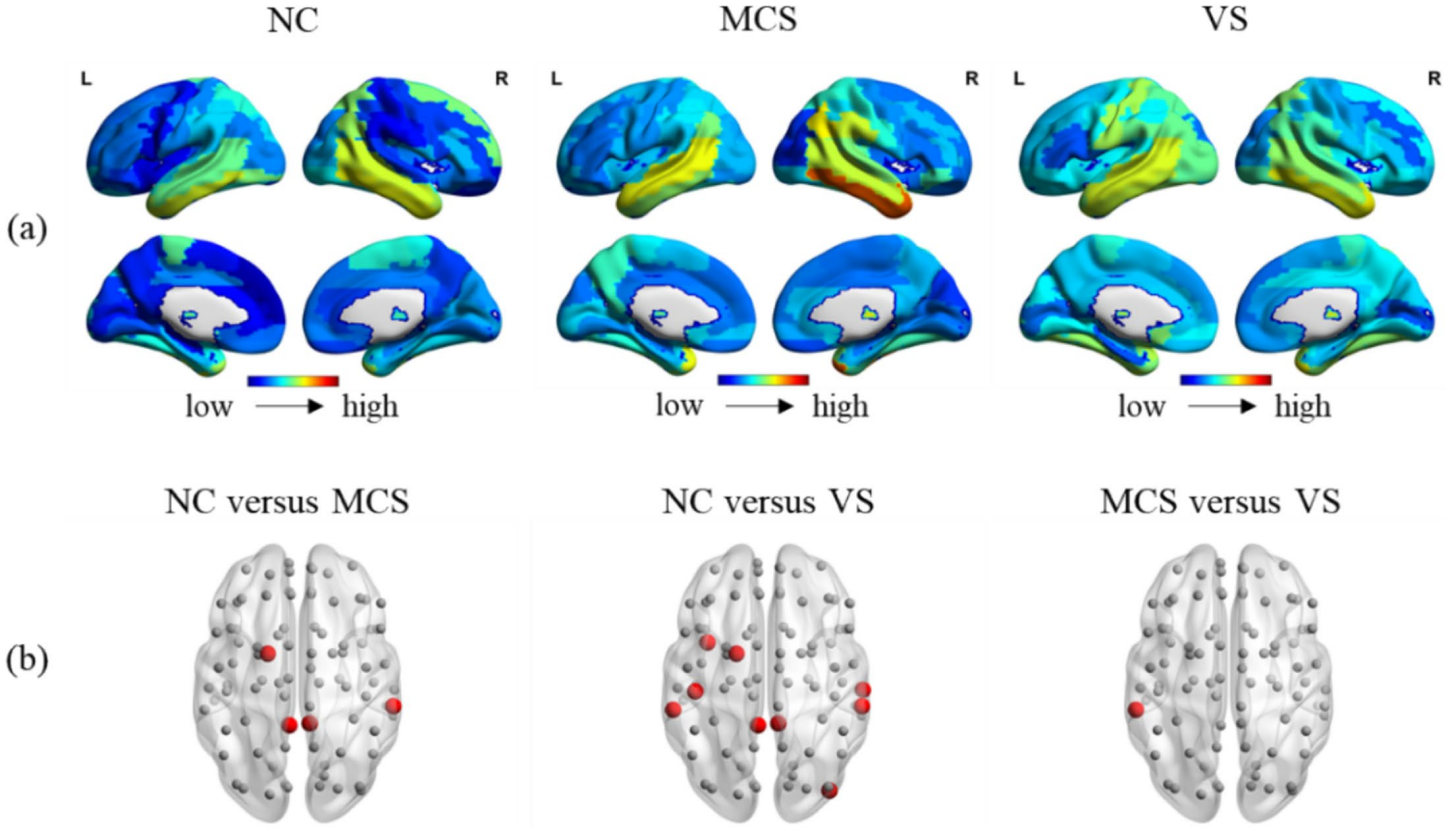
Between-group comparison in spatial variability. **(A)** The spatial variability across brain regions in the normal population, MCS patients and MCS patients; **(B)** between-group comparison in spatial variability. The brain regions with increased spatial variability (MCS > NC, *VS* > NC, and *VS* > MCS) are marked with larger red nodes The brain regions with no significant differences in spatial variability are marked with smaller gray nodes.

Brain is essentially organized into a network with spatio-temporal interaction characteristics. A meticulous investigation into the temporal and spatial variability may reveal the effects of DoC on this property of brain. Figures 7A, 8A showed the temporal and spatial variability across brain regions for each group. Figures 7B, 8B showed the differences in temporal and spatial variability between normal population and MCS patients, normal population and *VS* patients, and both groups of patients. The smaller gray nodes and larger red nodes indicate similar meanings as before. We identified an increase in temporal and spatial variability of multiple brain regions in MCS patients and *VS* patients in comparison with the normal population. Specifically, the alterations in temporal variability of MCS patients occurred within AUN, CON, DMN, SCN, FPN, SN, and visual network (VN), while the alterations in spatial variability of MCS patients occurred within DMN, SCN, and AUN. The alterations in temporal variability of *VS* patients occurred within AUN, CON, DMN, SCN, SN, and VN, while the alterations in spatial variability of *VS* patients occurred within DMN, SCN, SMN, VN, SN and AUN. Notably, increased temporal variability of the thalamus was detected in MCS patients and *VS* patients. With regard to the two groups of patients, we observed increased temporal and spatial variability in brain regions within AUN in *VS* patients. More details on the changes in temporal and spatial variability were provided in Supplementary Tables S3.

## 4 Discussion

Here, we expanded the detailed results to provide a nuanced and comprehensive understanding of the altered brain connectivity patterns in DoC patients, thereby shedding light on potential avenues for further research and clinical interventions.

MST has been confirmed to be a critical backbone for global information transmission. Between-group comparison in average coupling strength obtained using static MSTs showed consistent findings with the previous studies based on original network (Wang et al., 2018). This provides some evidence that static MST method is reliable in disentangling network-level dysregulations for DoC like original network. In addition, the attenuation in coupling strength and the disruption or alteration in functional connectivity may point to widespread alterations in neural communication of MCS patients and *VS* patients.

The topological analysis (Figure 3) reveals several intriguing features. In healthy brains, hub nodes identified based on static MSTs are much the same as those found based on original network (van den Heuvel et al., 2018; Oldham and Fornito, 2019). Once again, the reliability of MST-based analysis was verified. Despite the fact that static MST topology of MCS patients and *VS* patients is still an intermediate configuration between a line-like and a star-like MST structure, DoC resulted in a reorganization of the hub nodes. This directly affects the way brain regions interact with each other and may be an important reason for consciousness loss. Although the phenomenon was found in original network (Achard et al., 2012), it is the first time that the reorganization of the hub nodes in DoC patients is discussed based on MST. These preliminary observations were then further refined in the analysis of degrees.

Quantitative assessment of changes in topological structure is facilitated by the analysis of global and local measures (Figures 4, 5). The increased characteristic path length in MCS patients and *VS* patients potentially contributes to difficulties in information exchange between brain regions and indicates a diminished ability for functional integration (Rizkallah et al., 2019; Chennu et al., 2017). Previously, we also observed this alteration in original network (Yang et al., 2023). This illustrates that, despite discarding many details, the essential changes in network structure caused by DoC are still preserved on the MST. Similarly, as a measure of distance, an increase in diameter underscores the challenges in efficient information transmission between remote brain regions. As a side note, we detected a larger diameter in *VS* patients than in the normal population. However, FDR correction diluted this difference so that in the end only MCS patients had larger diameters than the normal population. Definitely, the information transmission is most efficient in the normal population, even between remote brain regions. The increased leaf fraction implies some nodes are less important in MCS patients and *VS* patients. Although different, the tree hierarchy in the normal population and DoC patients ranges from 0 to 0.5, which quantitatively confirms that their static MSTs were intermediate configurations.

Earlier, we explore hub nodes using nodal degree. Statistical tests showed that degree of the brain regions situated within FPN, DMN and AUN was significantly different between the normal population and MCS patients. This suggests that the reorganization of hub nodes in MCS patients may occur in FPN, DMN and AUN. Similarly, the reorganization of hub nodes in *VS* patients may occur in DMN and SMN. The betweenness centrality of a node quantifies its ability for communication control over other brain regions. Compared to the normal population, we detected changes in this ability of brain regions within FPN, AUN and DMN in MCS patients, and of brain regions located in CON and DMN in *VS* patients. The eccentricity of a node quantifies the transmission efficiency between that node and the most distant node reachable by it. The increased eccentricity of brain regions within FPN, AUN, SMN, DMN, CON, and SN in MCS patients, and of brain regions within CON, FPN, DMN and SCN in *VS* patients indicates a slower information transmission between these brain regions and the most distant brain regions reachable by them. Resting-state networks such as DMN, FPN and SN are intricately linked to cognitive control (Menon and D’Esposito, 2022; Menon, 2023) and consciousness (Vincent et al., 2007; Horovitz et al., 2008; Larson-Prior et al., 2009; Demertzi et al., 2014; Wu et al., 2015; Qin et al., 2015; Fischer et al., 2016). These changes in local measures highlight the intricate nature of the impact of DoC on specific resting-state networks.

Even though brain activity changed dynamically over time, the spatial distribution of hub nodes was relatively stable across multiple epochs (~100 s) within a continuous state (Figure 6). This stability is well supported by previous test–retest reliability studies (Tomasi et al., 2017; Mueller et al., 2015; Noble et al., 2017; Noble et al., 2017) and dynamic brain network-based research (Li et al., 2020), emphasizing the robustness of higher-order association regions, especially those within DMN. Exploring the factors contributing to this stability, even in the case of impaired consciousness in MCS patients and *VS* patients, opens avenues for exploring the resilience and adaptability of the human brain. However, our current understanding about this stability of functional organization is very limited.

Our findings support increased temporal variability in brain regions belonging to AUN, CON, DMN, SCN, FPN, SN, and VN in MCS patients, and in brain regions belonging to AUN, CON, DMN, SCN, SN, and VN in *VS* patients. Temporal variability sheds light on the extent to which the connectivity patterns of specific brain regions change over time. The high temporal variability suggests strong node flexibility and unstable connectivity patterns. The increased spatial variability was found in brain regions belonging to DMN, SCN, and AUN in MCS patients, and in brain regions belonging to DMN, SCN, SMN, VN, SN and AUN in *VS* patients. The spatial variability portrays the similarity in the time sequences of all functional connectivity linked to specific brain region. The high spatial variability indicates that all functional connectivity sequences associated with this brain region were fairly independent. This phenomenon is not unique to DoC. Previous study has revealed lower temporal variability in DMN regions in patients with schizophrenia, but higher temporal variability in DMN regions in patients with autism/attention deficit hyperactivity disorder (Zhang et al., 2016). The increased temporal and spatial variability in specific brain regions within critical resting-state networks of MCS patients and *VS* patients may provide a new clue to explaining consciousness loss.

Generally, the integrity of thalamo-cortical and cortico-cortical connectivity is considered to be necessary for the existence of awareness. The thalamus’s role as a gateway for transmitting sensory signals to the cortex underscores its importance in maintaining wakefulness and awareness (Lemaire et al., 2022; Malekmohammadi et al., 2019). The increased temporal variability in thalamus may add a new perspective to our understanding of altered consciousness in MCS patients and *VS* patients.

We identified that the hub nodes of MCS patients and *VS* patients are somewhat differentiated. The analysis of nodal degree suggests that MCS patients and *VS* patients may have differential hub nodes within the FPN. Interestingly, we again identified brain regions within FPN in MCS patients have a stronger ability in communication control over other brain regions than in *VS* patients. Several studies have shown differences in brain activity in the frontoparietal region between MCS patients and *VS* patients. This supports our findings to a certain extent. The observed difference in temporal and spatial variability between MCS patients and *VS* patients was reflected in AUN. MCS patients have a higher level of auditory and language function compared to *VS* patients. The differences in temporal and spatial variability within AUN between MCS patients and *VS* patients may benefit from this. Overall, the differences in functional connectivity between the two groups of patients focused on the node level. Despite the similarity of brain activity at the global level between MCS patients and *VS* patients (a slower transmission efficiency, a decreased ability for functional integration, etc.), differences in functional connectivity at the nodal level may provide some explanation for the preservation of limited consciousness in MCS patients.

Acknowledging the limitations of the study is crucial for a comprehensive interpretation of the findings. All our findings are still obtained based on a dataset with a small sample size. A small sample size and the high individual variability may introduce challenges in achieving robust statistical findings. Future research should focus on a dataset with more adequate samples to enhance the generalizability of the observed patterns. Furthermore, the utilization of the AAL brain template for dividing brain regions may introduce inaccuracies in matching resting-state networks, such as DMN, FPN and SN, etc. The research incorporating more refined brain templates and advanced neuroimaging techniques may well capture more nuanced changes.

## 5 Conclusion

In summary, our in-depth discussion delves into various facets of the results, emphasizing the multifaceted impact of DoC on functional connectivity. This study not only furthers our understanding of the neural mechanism underlying impaired consciousness but also may identify potential markers for diagnostic and therapeutic exploration. The limitations outlined provide valuable insights for the design of future studies, ensuring a more comprehensive exploration for DoC.

## Data Availability

Data are not publicly available but are available from the corresponding author on reasonable request.
