# Supplementary material for: Deciphering network dysregulations and temporo-spatial dynamics in disorders of consciousness: insights from minimum spanning tree analysis
Source: Front Psychol. 2024 Dec 19;15:1458339. doi: 10.3389/fpsyg.2024.1458339 (PMC11693494; doi:10.3389/fpsyg.2024.1458339)
Supplement: Supplementary file 4 [file Data_Sheet_1.docx]

Supplementary Material

Deciphering Network Dysregulations and Temporo-Spatial Dynamics in Disorders of Consciousness: Insights from Minimum Spanning Tree Analysis

Yangyang Dai^1†^, Qiheng He^2†^, Shan Wang^6^, Tianqing Cao^2^, Xiaoke Chai^2^, Nan Wang^2^, Yijun Dong^7^, Peiling Wong^8^, Jianghong He^2^, Feng Duan^1*^, Yi Yang^2, 3, 4, 5*^

*** Correspondence:**
Feng Duan, duanf@nankai.edu.cn
Yi Yang, yangyi_81nk@163.com

(a) (b)

(c)

Figure S1. The spatial distribution of hub nodes within each time window. (a) The normal population; (b) MCS patients; (c) VS patients. The small yellow squares in each row indicate the hub nodes in the current time window.
